# Electricity consumption variation versus economic structure during COVID-19 on metropolitan statistical areas in the US

**DOI:** 10.1038/s41467-022-34447-7

**Published:** 2022-11-19

**Authors:** Jinning Wang, Fangxing Li, Hantao Cui, Qingxin Shi, Trey Mingee

**Affiliations:** Department of Electrical Engineering and Computer Science, The University of Tennessee, Knoxville, TN 37996 USA

**Keywords:** Energy modelling, Electrical and electronic engineering

## Abstract

The outbreak of novel coronavirus disease (COVID-19) has resulted in changes in productivity and daily life patterns, and as a result electricity consumption (EC) has also shifted. In this paper, we construct estimates of EC changes at the metropolitan level across the continental U.S., including total EC and residential EC during the initial two months of the pandemic. The total and residential data on the state level were broken down into the county level, and then metropolitan level EC estimates were aggregated from the counties included in each metropolitan statistical area (MSA). This work shows that the reduction in total EC is related to the shares of certain industries in an MSA, whereas regardless of the incidence level or economic structure, the residential sector shows a trend of increasing EC across the continental U.S. Since the MSAs account for 86% of the total population and 87% of the total EC of the continental U.S., the analytical result in this paper can provide important guidelines for future social-economic crises.

## Abstract (web summary)

In this paper the authors construct estimates of electricity consumption changes at the metropolitan level across the continental U.S., during the initial two months of the COVID-19 pandemic, demonstrating variations in electricity consumption patterns based on several factors.

## Introduction

COVID-19 broke out and spread rapidly in the U.S., compelling human society to reduce activities involving physical contact. Enforcement of shelter-in-place orders in many states led to transformations in people’s working and living styles, such as the rise of the work-from-home model and decreased commuting needs^1^. As a result, the demand for energy resources, such as gasoline, jet fuel, coal, and natural gas, experienced a sharp decrease^2–4^. The drastic minimization of human activities also impacted the environment, both positively and negatively. While greenhouse gas emissions underwent a dramatic decline^5–7^ and air quality, beach cleanliness, and environmental noise levels improved, increased waste, especially medical waste, was recognized as a challenge^8,9^. The electric power industry was also significantly impacted during the pandemic, and that impact will be the topic of this work.

On the electricity generation side, the share of renewable power generation has increased continually during the COVID-19 pandemic^10–12^. This is due primarily to policy support and the continuously decreasing cost of renewables despite lags in the supply chain and delays in the deployment process^13^. Meanwhile on the electricity demand side, total electricity consumption (EC) decreased and EC composition changed^14^, with the daily peak demand decreasing and arriving during later hours^15^. Power infrastructure maintenance was also affected due to supply chain disruptions^16^. To secure both the power supply and their employees’ health, the electric power industry overall reacted rapidly and effectively by encouraging employees to work from home, monitoring employee health conditions, and extending employee shift times to reduce infection^16^. Despite the clear overall trends within the electric power industry, the implications of the pandemic on the power grid differ from region to region across the continental U.S. For example, while a significant reduction in demand occurred in the midcontinent area, the electricity demand in Florida remained almost unchanged^17^. In addition, the sensitivity of total demand to the mobility of the retail sector has varied between cities^18^. However, detailed shifting patterns of nationwide EC are not available. Because EC and economic production are frequently linked, it is well known that the gross domestic product (GDP), as an index of economic production, can forecast EC^19–22^. However, EC is not only linked to economic output, but also to economic structure, which can affect the EC projection^23,24^. In other words, changes in the economic structure can cause shifts in EC^25,26^. This study considers each metropolitan statistical area (MSA) as the basic unit and explores the connection between economic structure and EC shift patterns following the beginning of the pandemic in the U.S. In summary, county-level EC has been calculated using the GDP, population, and state-level EC data. Then MSA-level EC estimates are aggregated from county-level EC data. The estimates cover 380 MSAs in the continental U.S. out of the total 384 MSAs rigorously defined by the United States Office of Management and Budget. EC estimates for the remaining 4 MSAs located in Hawaii and Alaska were not calculated. These 380 MSAs account for 86% of the total population and 87% of the total EC of the continental U.S., while the rural areas account for 14% of the total population and 13% of the total EC. Thus, understanding the EC patterns and economic structures of these MSAs is of great importance. The studied time periods are April–May 2019 and April–May 2020, and the data for these time periods includes total EC and residential sector EC. The April–May time period was selected because the first two months of the pandemic in the U.S. are critical to understanding the pandemic impact on EC, as there was no preparation or organized response to such a social-economic emergency. The detailed data resources and analysis procedures are discussed in the “Methods” section.

The metropolitan-level perspectives in this study help to demonstrate the connection between EC and economic structure because MSAs accommodate a high population density and integrate sizeable industries.

Based on the above motivations, this paper constructs estimates of metropolitan level EC variation from April–May 2019 to April–May 2020 in the U.S. Here, we show that there is an evident pattern shift of total EC, and the patterns are different for different economic structures in different metro areas. Meanwhile, although there is a nominal residential load increase of a few percentage points across all pandemic incidence levels and economic structures, the amount of residential load increase is not related to particular levels of pandemic severity or particular economic structures.

## Results

### COVID-19 incidence map on the metropolitan level during April–May 2020

After the first COVID-19 case was confirmed in the state of Washington in January 2020, it spread throughout the U.S. at an unexpected speed. Most states issued stay-at-home orders before the end of March, shutting down unessential places to restrain the pandemic’s spread. The pandemic entered a plateau in the U.S. during April and May. However, when it came to the end of May, the reopening process and mass gathering activities accelerated pandemic spread and increased EC. To analyze the impact of stay-at-home orders on EC during the pandemic, this paper specifies the time window between 1 April and 31 May 2020. The information from these first two months represents initial and unprepared responses to the COVID-19 pandemic, and thus has the most significant implication for a similar future social-economic emergency leading to a lockdown.

**Fig. 1:**
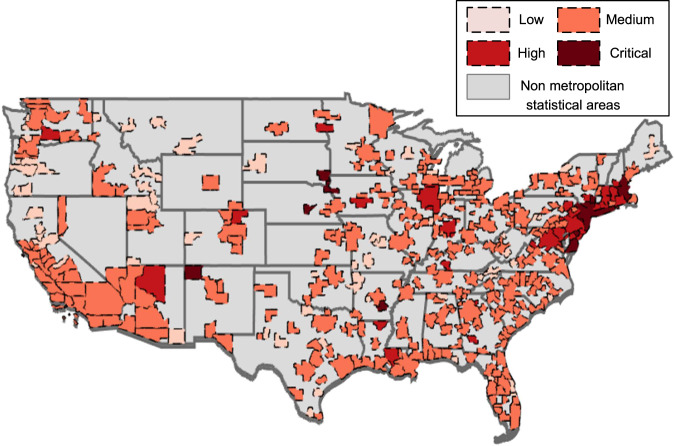
COVID-19 incidence map. COVID-19 Incidence map of 380 metropolitan statistical areas in the continental U.S. during April–May 2020.

Figure 1 shows the COVID-19 incidence map of the 380 MSAs in the continental U.S. during the two-month window from April to May 2020. The COVID-19 incidence of MSAs are calculated based on Eq. (2) (see the “COVID-19 incidence level calculation” subsection of the “Methods” section for incidence level calculation) and plot on the map from U.S. Census Bureau^27^. The pandemic situation on the west coast (e.g., the states of Washington, Oregon, and California) was low to medium, and the situation along the southeastern coast (e.g., the states of North Carolina, South Carolina, Georgia, and Florida) was medium. However, states along the northeastern coast were experiencing high-to-critical levels of COVID-19 incidence. The largest critical area was the MSA of New York-Newark-Jersey City, NY-NJ-PA, which encompasses 20 million people. Along the northeastern coast, there were two other MSAs at critical incidence levels: Vineland-Bridgeton, NJ and Salisbury, MD-DE. The incidence level map shows geographical relevance among these areas since the adjacent MSAs to New York-Newark-Jersey City, NY-NJ-PA also experienced a high incidence of COVID-19 at this time.

### Economic structure features

**Fig. 2:**
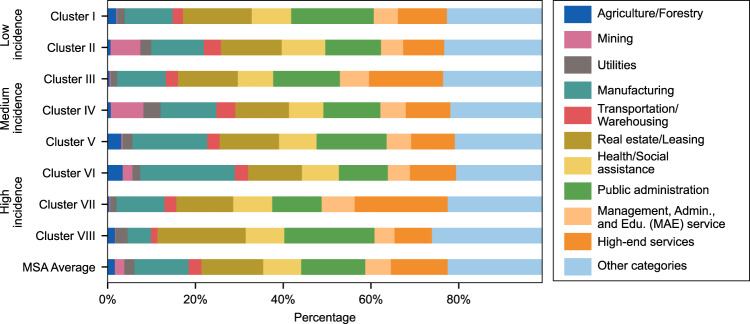
Cluster centers of MSA economic structure. The length of each colored bar represents the GDP percentage of the corresponding economic category.

Based on the economic structure described by the 20 selected GDP-related variables, the areas of different incidence levels are categorized into separate clusters, respectively (See “Economic structure clustering analysis” subsection in the “Methods” section). Figure 2 shows the economic structure of cluster centers of low (Cluster I and II), medium (Cluster III, IV, and V), and high incidence level (Cluster VI, VII, and VIII) MSAs. It should be noted that a cluster center is calculated from the mean value of all the observations in the corresponding cluster. For simplicity, Fig. 2 shows the categories that demonstrate statistically significant difference among each cluster (i.e., difference from MSA averages), while all other categories with no significant difference among different clusters are combined into “Other categories.” Further, GDP categories are rearranged as follows: (1) management, administrative, and educational services are combined as “MAE service”; (2) information, finance/insurance, and professional services are combined as “high-end services”; and (3) the “Other categories” include construction, wholesale trade, retail trade, accommodation/food services, arts/entertainment/recreation, and other services. There are 11 MSAs in critical COVID-19 incidence level with no significant clustering trends, so the clustering results from these 11 MSAs are not discussed in the economic clustering analysis below. For comparison, the average economic composition of all 380 MSAs is displayed as different sections in the stacked bar chart in Fig. 2. The clusters’ unique economic characteristics are summarized as follows.

- In terms of agriculture/forestry, Cluster V and VI have a higher percentage when compared to the MSA average level. Furthermore, one can also observe that the share of manufacturing of these two clusters is larger than the average level.
- Regarding the mining industry, Cluster II and IV have a higher proportion than the MSA average level. Similarly, the transportation/warehousing of these two clusters is also above the average level.
- As for real estate/leasing, Cluster I and VIII have a higher share than the MSA average. Also, their percentage of public administration is greater than the MSA average.
- Another noteworthy point is that Cluster III and VII have a higher percentage of high-end services and MAE services.
- The MSA average is shown by the last bar of the figure for easy comparison.

### EC variation on the metropolitan level after COVID-19

Following the initial outbreak of COVID-19, the stay-at-home trend led to fewer human activities in industrial and commercial sectors. Therefore, total EC experienced a remarkable decrease, while residential EC enlarged widely since people stayed at home for much longer periods of time than usual.

**Fig. 3:**
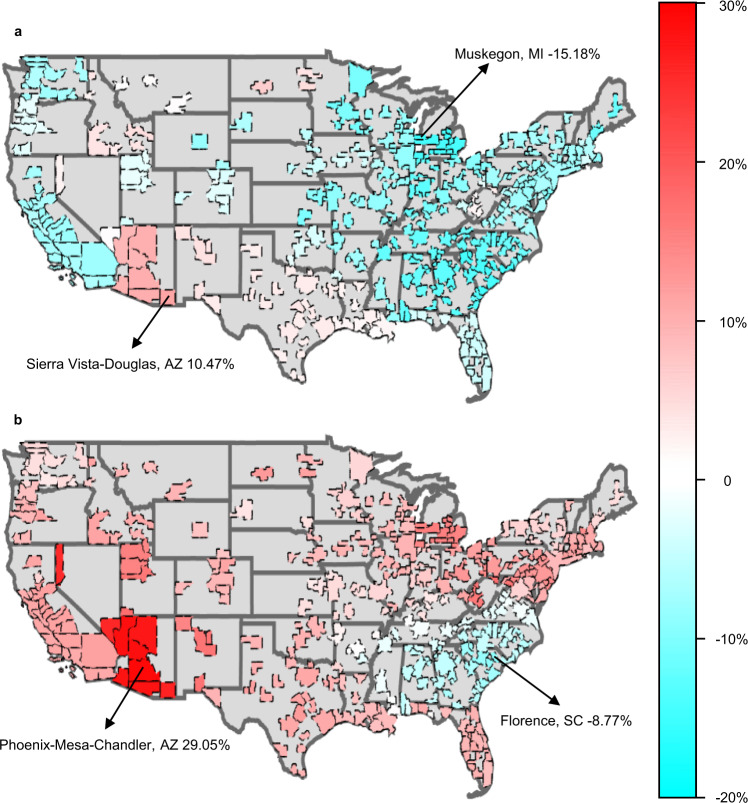
Spatial heterogeneity in EC change on metropolitan level after COVID-19. **a** Total EC change. **b** Residential EC change.

Figure 3 shows the EC change in the U.S. on the metropolitan level after the pandemic began. The EC variation of MSAs are estimated (See “EC estimates” subsection in the “Methods” section) and plot on the map from U.S. Census Bureau^27^. It shows the overall trend that total EC declined while residential EC increased, which is reasonable due to the implementation of the work-from-home model, although some regions experienced the opposite change.

Figure 3a illustrates the total change in EC at the metropolitan level in the April–May two-month window in both 2019 and 2020. It can be seen that the electricity demand shrinks in most regions of the country. The sharpest decline (−15.18%) occurred in Muskegon in the state of Michigan, and other MSAs in Michigan also experienced more than a 12% decrease in total EC, where the 95% confidence interval (CI) of the average value is [−14.67%, −13.89%], *n* = 15, and the alpha used here is 0.95, which is the default threshold value in the rest of this paper. Similarly, MSAs in the midwestern states (Illinois, Indiana, Iowa, Kansas, Michigan, Minnesota, Missouri, Nebraska, North Dakota, Ohio, South Dakota, and Wisconsin) decreased by about 8.88% in total EC, where the 95% CI is [−9.71%, −8.06%], *n* = 96. It should be noted that there are many different definitions of U.S. regions by various government agencies, and the regions in this paper are loosely defined for illustrative purpose. Total EC decreases in the northeast (New York, Connecticut, Maine, Massachusetts, New Hampshire, New Jersey, Pennsylvania, Rhode Island, and Vermont) were about 7.45%, where the CI is [−7.89%, −7.01%], *n* = 51. On the west coast, the decreasing patterns in the state of Oregon (CI = [−2.13%, −1.33%], *n* = 8) were lighter than in the state of California (CI = [−7.13 %, −6.63 %], *n* = 26) and the state of Washington (CI = [−7.08%, −4.04%], *n* = 13). Decreases in the southeastern and nearby states (Alabama, Florida, Georgia, Kentucky, Maryland, Mississippi, North Carolina, South Carolina, Tennessee, Virginia and West Virginia) were also somewhat significant (CI = [−9.69%, −8.07%], *n* = 114). Meanwhile, MSAs in Florida saw notably smaller decreases in total EC, where the CI is [−3.65%, −3.17%], *n* = 22.

Despite the overall decrease in total EC, some MSAs in the south consumed more electricity after the pandemic took hold. The total EC of MSAs in the states of Louisiana, Texas, and New Mexico increased slightly, where the CIs are [0.69%, 1.21%], [1.32%, 2.14%], and [2.70%, 3.51%], respectively, and the sample sizes are 9, 25, and 4, respectively. MSAs in the state of Arizona consumed much more electricity, where the CI is [8.59 %, 9.85%], *n* = 7. The largest increase in total EC occurred in Sierra Vista-Douglas, AZ (10.47%). The total EC of regions in the states of North Dakota, Idaho, and Nevada also increased slightly.

In contrast, Fig. 3b depicts the variation of residential EC on the metropolitan scale between April–May 2019 and April–May 2020. Nationally, the residential sector saw an increasing trend. The largest increase in residential sector EC occurred in Phoenix-Mesa-Chandler, AZ (29.05%). Other MSAs in Arizona and Nevada also experienced remarkable expansions of more than 20% in residential EC, where the 95% CIs are [26.88%, 28.65%] and [24.30%, 25.92%], respectively, and the sample sizes are 7 and 3, respectively. MSAs in other southern states of New Mexico, Texas, Louisiana, and Florida also increased largely in the residential sector, where the CIs are [14.25%, 17.23%], [8.83%, 9.95%], [7.23%, 8.04%], and [7.53%, 9.23%], respectively, and the sample sizes are 4, 25, 9, and 22, respectively. The northeastern regions (New York, Connecticut, Maine, Massachusetts, New Hampshire, New Jersey, Pennsylvania, Rhode Island, and Vermont) also experienced a large expansion in residential EC, where the CI is [8.32%, 10.18%], *n* = 51. Similar increases in residential sector EC were also observed in California and Oregon, where the 95% CIs are [9.71%, 10.73%] and [7.61%, 9.38%], respectively, and the sample sizes are 26 and 8, respectively. In contrast, the increases in the state of Washington were moderate, where the CI is [3.40%, 4.88%], *n* = 13. Similarly, slight increases of residential EC occurred in the central U.S. regions (e.g., the states of Utah, Colorado, Kansas, Oklahoma, Missouri, Illinois, Indiana, and Kentucky), where the aggregated CI for these eight states is [5.93%, 7.80%], *n* = 60, and each individual state’s variation pattern is similar, as shown in the colored map in Fig. 3b. Also, we may observe a few transitional states such as Virginia, where the residential sector EC reduced very slightly, with the CI being [−1.62%, −0.81%] (*n* = 7) which is lower than the CIs of the northeastern states and higher than the southeastern states; for example, residential EC dropped by no more than 1% in Richmond, VA. Note, the above discussion lists a few high-level patterns observed as examples, while it does not necessarily cover all MSAs in every state. More details can be found in the data set provided in the “Data Availability” section.

By comparison, residential EC in parts of the southeastern region changed in the opposite direction. Decreases occurred in North Carolina, South Carolina, Georgia, and Alabama, where the CI is [−5.25%, −4.42%], *n* = 51. The largest reduction occurred in Florence, SC which decreased by 8.77% in the residential sector. The results show EC changes across the U.S. at the metropolitan level. It can be observed that although EC variation patterns differed from region to region, the overall trend in EC was towards a decrease in total EC and an increase in residential EC.

### EC variation and economic structure

Economic structure reflects the industry and commercial components of a specific area, impacting EC. As such, reductions in EC caused by lockdown policies are interconnected with the economic structure. For example, New York Independent System Operator (NYISO) observed that the decline in electricity demand in the state of New York is mainly attributed to reduced commercial sector consumption^3^.

**Fig. 4:**
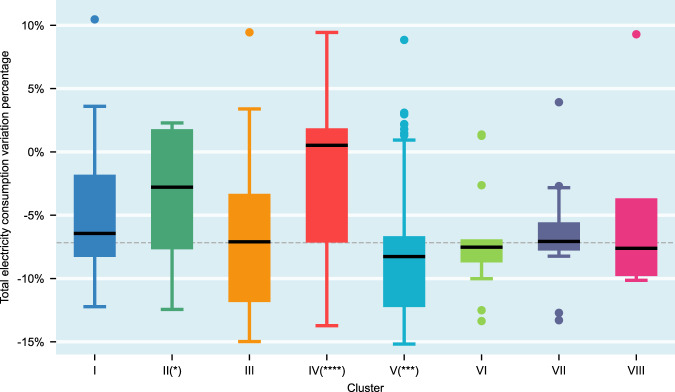
Total EC variation among different clusters. Box and whisker plots of total EC variation of April–May 2020 in comparison to pre-pandemic level; boxes depict the upper and lower quartiles of the data; black solid lines depict the median values; whiskers depict the range of the data excluding outliers (outliers are defined as observations larger than 1.5× the inter-quartile range from the upper or lower quartiles); the grey dashed line depicts the median value of the total EC variation of 380 MSAs. Two-sided Wilcoxon rank sum tests of each Cluster versus all the MSAs are performed, and *n*_2_ = 380 which is the case for all the eight tests. Cluster I: *p* = 0.0709, *n*_*1*_ = 44, *W* = −1.8063; Cluster II: *p = 0.0116, *n*_*1*_ = 19, *W* = −2.5246; Cluster III: *p* = 0.4784, *n*_*1*_ = 110, *W* = 0.7088; Cluster IV: ****p < 1e-4, *n*_*1*_ = 42, *W* = −4.2970; Cluster V: ***p = 0.0001*, n*_*1*_ = 120, *W* = 3.8057*;* Cluster VI: *p* = 0.6487*, n*_*1*_ = 13, *W* = 0.4556; Cluster VII: *p* = 0.8865*, n*_*1*_ = 16, *W* = −0.1427; Cluster VIII: *p* = 0.8476, *n*_*1*_ = 5, *W* = −0.1921.

Figure 4 shows the boxplot of total EC variation between April–May 2019 and April–May 2020 on the metropolitan level among different economic structure clusters. It clearly demonstrates an overall pattern of total EC reduction across all clusters. If we connect Figs. 4, 2 to build some connections between total EC reduction and economic structures, the following observations can be presented.

- The total EC change indicates that Clusters II and IV have significantly higher EC reduction than the average. Both of them have a sizable mining industry (about 7%) while other economic categories are similar to the MSA average, as shown in Fig. 2. Thus, it can be inferred that MSAs with a high proportion of mining industry saw less EC reduction than other MSAs (i.e., mining industry EC is less affected during the pandemic), which is evidenced by a statistical difference in total EC reduction of II-and-IV versus other MSAs (Wilcoxon rank sum test: ****p < 1e-4, *n*_*1*_ = 61, *n*_*2*_ = 319, *W* = 5.6761). This is reasonable, because the mining industry forms a significant portion of total electricity demand.
- Another significant observation is that both Clusters V and VI have a significantly higher proportion of agriculture/forestry and manufacturing than the MSA average, while their other economic categories are similar to the MSA average. The total EC of both clusters seem to have greater declines than the average level of total EC (i.e., the grey dashed line in Fig. 4), so this shows that agriculture/forestry and manufacturing tend to have more EC reduction during pandemic than other categories. Further observation is that the total EC of Cluster VI has less reduction than Cluster V, which can be possibly ascribed to higher mining industry share in Cluster VI than in Cluster V because mining industry EC is less affected during the pandemic, as discussed previously.
- Clusters III and VII share similar economic structure characteristics, with a concentration on intelligence-intensive services such as the economic category of high-end services (i.e., information, finance/insurance, professional services) and the category of MAE (i.e., management, administrative, and educational) services. However, the total EC of Clusters III-VII does not demonstrate statistically significant differences versus the total EC of other MSAs (Wilcoxon rank sum test: *p* = 0.3919, *n*_*1*_ = 126, *n*_*2*_ = 254, *W* = −0.8516). Thus, it can be statistically concluded that the load reduction in the high-end services and MAE services is aligned with average EC reduction. The possible reason is that although the computing loads of high-end and MAE services are shifted from offices to homes, and the residential home air conditioning loads stay at the same level before and after the pandemic, the air conditioning and lighting loads in commercial buildings should reduce considerably during the initial months of the pandemic. This makes the reduction pattern of high-end and MAE services similar to other economic categories.
- Both Cluster I and VIII feature a disproportionately high share of the real estate/leasing and public administration industries in their economic structure, where the total EC reduction for the combination of Cluster I and VIII is statically less than in other clusters (Wilcoxon rank sum test: *p = 0.0452, *n*_*1*_ = 49, *n*_*2*_ = 331, *W* = 2.0032). It means that the real estate business and public administration categories tend to have less reduction in total EC than other categories.
- Regarding the impacts of the pandemic, total EC changes among different incidence MSAs do not show an obvious pattern.

In summary, based on the observation, while there is an overall pattern of reduction in total EC across all clusters, the total EC variation is statistically related to economic structure during the initial months of COVID-19. More specifically, economic structures more dependent on the mining industry exhibit significantly less EC reduction than other categories, and real estate/leasing and public administration industries also demonstrate less EC reduction after the start of the COVID-19 pandemic. In contrast, agriculture/forestry and manufacturing-dependent economic structures exhibit more EC reductions than other categories. Further, the EC reduction of intelligence-intensive services (e.g., high-end services, MAE services) is not significantly different from other categories.

**Fig. 5:**
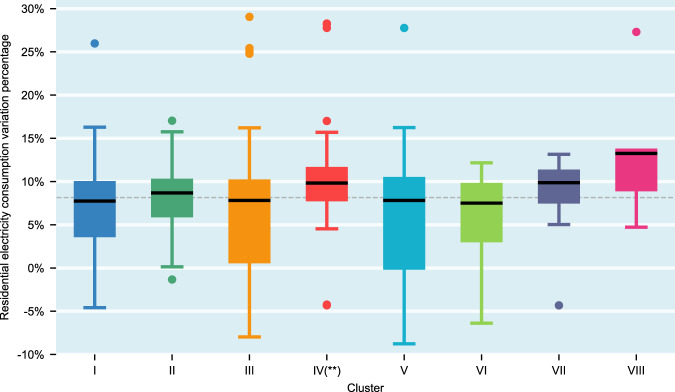
Residential EC variation among different clusters. Box and whisker plots of residential EC variation of April–May 2020 in comparison to pre-pandemic level in April–May 2019; boxes depict the upper and lower quartiles of the data; black solid lines depict the median values; whiskers depict the range of the data excluding outliers (outliers are defined as observations larger than 1.5× the inter-quartile range from the upper or lower quartiles); the grey dashed line depicts the median value of the residential EC variation of 380 MSAs. Two-sided Wilcoxon rank sum tests of each Cluster versus all the MSAs are performed, and *n*_2_ = 380 is the case for all eight tests. Cluster I: *p* = 0.5535, *n*_*1*_ = 44, *W* = 0.5926; Cluster II: *p* = 0.4344, *n*_*1*_ = 19, *W* = **−**0.7817; Cluster III: *p* = 0.3734, *n*_*1*_ = 110, *W* = 0.8901; Cluster IV: **p = 0.0023, *n*_*1*_ = 42, *W* = −3.0518; Cluster V: *p* = 0.0992, *n*_*1*_ = 120, *W* = 1.6488; Cluster VI: *p* = 0.6684, *n*_*1*_ = 13, *W* = 0.4283; Cluster VII: *p* = 0.0994, *n*_*1*_ = 16, *W* = −1.6477; Cluster VIII*: p* = 0.0648, *n*_*1*_ = 5*, W* = −1.8465.

Figure 5 shows the boxplot of residential EC variation between April–May 2019 and April–May 2020 at the metropolitan level among different economic structure clusters. It evidently demonstrates an overall pattern of residential EC increase across all clusters. The figure shows that the residential EC increase in Cluster IV is higher than the average level. However, no obvious reason can be concluded. Cluster VII and VIII are also well above the average level, but the difference is not statistically significant. The reason is that the small sizes of observations of Cluster VII and VIII result in statistical insignificance. Overall, the median values among other clusters were not significantly different, and the median values of residential EC increases of all clusters are ~7–10%.

In summary, total EC variation during the initial months of COVID-19 is shown to be mainly related to economic structure, whereas residential EC is shown to have increased regardless of economic structure and COVID-19 incidence level.

### Observation and verification from other reports

Metropolitan-level EC data across the nation is not available because the grid is operated by the power system operator which is across administrative divisions, and the reductions are reported among the service territory rather than each MSA. However, partial estimates of electricity demand reduction for regions can be verified by reports from the California Energy Commission (CEC) and other power grid operators such as the Midcontinent Independent System Operator (MISO) and PJM.

The CEC reported that, in California, average weekday total EC reduced by 9% in April 2020 compared to the same period in 2019^28^. In our estimation, the average MSA level reduction in total EC in California is 6.9%, where the 95% CI is [−7.1%, −6.6%], *n* = 26 in April–May 2020 compared to the same two-month period in 2019. Because the reduction on weekends is roughly 5%-10% lower than on weekdays^14,15^, the numbers from the CEC report are roughly aligned with our estimates. In addition, the CEC observed that, in California, the increase of the residential EC ranged from 8.9% to 12.4% for the five-month window of January-May 2020 in comparison to January-May 2019. In contrast, we estimated that the reduction in MSA demand in the residential sector in California was about 10.2% between April–May 2019 and April–May 2020, which is almost the middle of the CEC-reported range [8.9%, 12.4%], *n* = 26. Further, MISO, which covers most parts of 11 states in the midwestern U.S. and Manitoba in Canada, observed a 9.34% decrease in total EC during April–May 2020 as compared to April–May 2019^29^. In our estimates among the states in the MISO service territories (North Dakota, South Dakota, Minnesota, Iowa, Wisconsin, Michigan, Illinois, Indiana, Arkansas, Mississippi, and Louisiana), the average level of metropolitan reduction in total electricity demand is 8.0%, where the 95% CI is [−9.1%, −6.9%], *n* = 84. In addition, PJM, a regional transmission organization that operates electricity markets, reported about a 10%-14% decrease in the first half May 2020 and 6%-11% decrease between May 16 to June 3, 2020^30^. In our estimates, the MSAs in the PJM service territory (Delaware, Illinois, Indiana, Kentucky, Maryland, Michigan, New Jersey, North Carolina, Ohio, Pennsylvania, Virginia, West Virginia, and the District of Columbia) experienced a 9.3% reduction in total electricity demand during April–May 2020, where the 95% CI is [−10.1%, −8.8%], *n* = 118. Although the territories of MISO and the time-windows of the reports from the CEC and PJM are not exactly the same as in our estimates, our estimated reduction in total EC is essentially consistent with these reports.

In summary, the constructed metropolitan level electricity demand estimate is consistent with actual measurements from the CEC, MISO, and PJM. The reports from these operators confirm the credibility of our estimates of the EC variation on the metropolitan level for the two-month window of April–May in 2019 and 2020.

### Sensitivity analysis

The source data is critical to the results, which are updated and modified over time by the publisher. The COVID-19 data at the county level have been updated, resulting in changes in two MSAs out of a total of 380. GDP data at the MSA level were updated with 2019 data, which affects the economic structure of the MSAs. However, as shown in Supplementary Table 1, the gap between GDP categories within the same clusters is not significant.

The U.S. Energy Information Administration also updates the EC data at the state level, including the EC for 2020, which influences the EC estimates at the MSA level. As illustrated in Supplementary Table 2, the EC change with the updated data is relatively small in comparison to the previous version data. However, in the subsequent pattern analysis of EC variation, only Cluster V of residential EC changed from significant (*p = 0.0369, *n*_*1*_ = 104, *n*_*2*_ = 380, *W* = 2.0873) to insignificant (*p* = 0.0992, *n*_*1*_ = 120, *n*_*2*_ = 380, *W* = 1.6488), while the Wilcoxon rank sum tests of other clusters remained unchanged.

In summary, although the data were updated and modified during the development and revision of this paper, the analysis of the EC variation patterns persist with robustness. This further demonstrates the credibility and robustness of the EC patterns related to the clusters.

### Limitations

In this article, the nationwide estimates of EC on the metropolitan level in the U.S. are implemented with limited data. This limitation underlying the “Methods” section of this paper can be explored in the future: (1) The EC estimates rely on the assumption that the linear relationships between GDP-Total EC and Population-Residential EC are extrapolated from counties in California to other counties across the continental U.S. Although the linear relationship at the state level implies the effectiveness for the sum of the EC of all the counties in a state which is also the basis for the estimates of EC at the MSA level, its validity remains to be confirmed by other available county-level EC data. However, such data is not readily available at this time, and is difficult to collect. Also, in the extrapolation, uncertainties can be introduced by the degraded linearity between the county-level EC, GDP, and population in other states. Data transformation can be applied to assure the linearity. (2) The modeling of EC has drawn attention from many researchers and various methods have been proposed^31–34^. Although this article provides an easy-to-implement and effective way to estimate the EC on the MSA level, the accuracy of the estimation method will benefit from more data sources and more refined modeling methods, climate variables such as cooling-degree-days and other economic variables such as GDP per capita can be introduced as control variables to enhance the model of EC^35,36^. Further, a more comprehensive survey on the energy supply during the pandemic can lend from panel data analysis involving economics, electricity, petroleum, and gas^37,38^.

## Discussion

This paper proposes an easy-to-implement and effective method for estimating EC change under a widely applied lockdown policy, and reveals the connections between EC change and economic structure. By considering the economic features of regions as they relate to potential pandemics or other social-economic crises as a set of new regulation rules or constraints, power grid administrators can improve energy resource planning and power grid operation such that the future power systems will be pandemic-ready. Our EC change estimation method may potentially change the model of power grid constraints. A most recent example is the ongoing trend of incorporating of cyber-physical security (CPS) into power system operation and planning, in addition to classic physics-based security constraints. In other words, power grid constraint models may evolve from physics-only (conventional practices) to physics-and-CPS constraint models (as in some ongoing research works), and eventually to physics-CPS-and-pandemic all-inclusive constraint models (future studies). Thus, the impact of this work will be fundamental and substantial.

Our estimates of EC variation at the U.S. metropolitan level reveal the impacts of a large-scale lockdown policy following the outbreak of COVID-19 on both total EC and residential sector EC. Our estimates also show how EC variation is affected by the economic structure of different MSAs.

Our estimates indicate an overall decrease in total EC and an increase in residential sector EC. Although total EC decreased in most MSAs, the reduction amount differs from region to region. Based on in-depth analysis of economic structures, we have found that the reduction in total EC is related to the shares of certain industries in an MSA. High percentage shares in the mining industry and real estate/leasing are related to smaller decreases in total EC, whereas a large reduction in total EC is related to a high share of manufacturing. In contrast, regardless of the incidence level or economic structure, the residential sector shows a trend of increasing EC across the continental U.S. Seemingly, the increase in residential consumption was brought by the shelter-in-place orders issued during the April–May 2020 time period. Following the pandemic, some organizations may allow employees to work from home permanently, indicating that the pandemic may affect people’s lifestyles and society over a longer time scale than the temporary lockdown time. As a possible result, variations in both total and residential sector EC caused by the pandemic may never completely return to pre-pandemic levels.

The comparison of EC variation between different incidence levels is shown in Supplementary Table 3. One can observe that the total EC in lower incidence MSAs experienced less of a decrease than the MSAs in higher incidence levels, whereas the change in residential EC between MSAs at different incidence levels was not significant. Another interesting observation is that the correlation between COVID-19 incidence level and EC varies with respect to time. In the April–May time window at the state level, the Pearson coefficients between COVID-19 incidence and EC (total, residential) increased from (0.21, 0.22) to (0.36, 0.40), respectively, whereas the Pearson coefficients between COVID-19 deaths and EC (total, residential) increased from (0.17, 0.18) to (0.23, 0.24), respectively. This indicates that the relationship between EC and the pandemic is dynamic rather than static. Although the coefficients were not high in the early stage of the pandemic as the COVID-19 virus spread, EC can be viewed as another metric of the pandemic.

## Methods

### COVID-19 incidence level calculation

The incidence is a measure of epidemiological spread rate, as given by the following equation:

1 $$I=\frac{C}{P/100,000}$$

in which *I* is the incidence, *C* is the daily new confirmed cases, and *P* is the population. The seven-day moving average is applied when calculating *C* to eliminate the statistic’s fluctuation between weekdays and weekends. The four levels of COVID-19 are defined as:^39^

2 $$I=\left\{\begin{array}{ll}0,\hfill&{{{{{\rm{Low}}}}}}\hfill\\ \left[1,\,10\right),\hfill&{{{{{\rm{Medium}}}}}}\hfill\\ \left[10,\,25\right),\hfill&{{{{{\rm{High}}}}}}\hfill\\ \left[25,\,+\!{{\infty }}\right),&{{{{{\rm{Critical}}}}}}\hfill\end{array}\right.$$

The populations of MSAs were aggregated from 2019 county annual resident population estimates^40^. COVID-19 case data for each MSA was aggregated from U.S. county COVID-19 case data^41^. The incidence of COVID-19 on the metropolitan level was then calculated by Eq. (1). During the 61 days from 1 April to 31 May 2020, the most frequent incidence level was chosen as the metropolitan incidence level.

### Economic structure clustering analysis

GDP data from 2019^42^ is used to represent the pre-pandemic economic structure, and the missing values of 2019 were filled in using the data from 2015 to 2018. There are 35 lines of data in each MSA, with each line accounting for a category. However, 20 categories listed in Supplementary Table 4 were selected as the representative variables to address the overlap in the source data.

In some cases, there are missing values introduced from part of the data being hidden by the Bureau of Economic Analysis to avoid disclosing confidential information, such as Agriculture/Forestry from 2018 to 2019 in Supplementary Table 4. To address the missing values, the data were processed in four steps: (1) If the categories have valid observations within the most recent four years (2015-2018), the missing values were filled in with the average value of the valid values; (2) The GDP data of 2019 were scaled into percentage between 0 and 1 from quantity; (3) Regarding the categories that all five observations are absent, they are filled with $(1-s)/n$, where *s* is the sum of the non-zero categories and *n* is the number of missing categories; and (4) The scaled GDP data suffered from skewness to the right that can degrade the further clustering analysis. Therefore, a fifth root transformation was applied to alleviate the skewness issue. The data from Asheville, NC is given in Supplementary Table 4 as a snapshot of the source and preprocessed data.

Given the high dimensionality of the economic structure data, k-means^43^ was used for clustering. More details can be found in Supplementary Note 1. The distance metric used in this study is the Euclidean distance, and the elbow method is used to determine the number of clusters. Clustering analysis of economic structure, which can be used to classify MSAs according to their economic characteristics, will be used to further investigate the EC variation patterns in this paper.

### EC estimates

The estimates can be done in two steps which are described as follows.

In the first step, we used GDP and population as indicators of total and residential EC respectively. EC is categorized into four sectors: residential, commercial, industrial, and transportation. This study analyzed the variation of total EC, residential EC, and the proportion of the residential sector. However, EC data on the metropolitan level is not directly available. Therefore, estimates were constructed for further study.

Metropolitan level EC, including total consumption and residential consumption, was estimated through EC data at the state level from the U.S. Energy Information Administration (EIA). First, the total and residential data on the state level were broken down into the county level. Second, metropolitan level EC estimates were aggregated from the counties included in a given MSA.

**Fig. 6:**
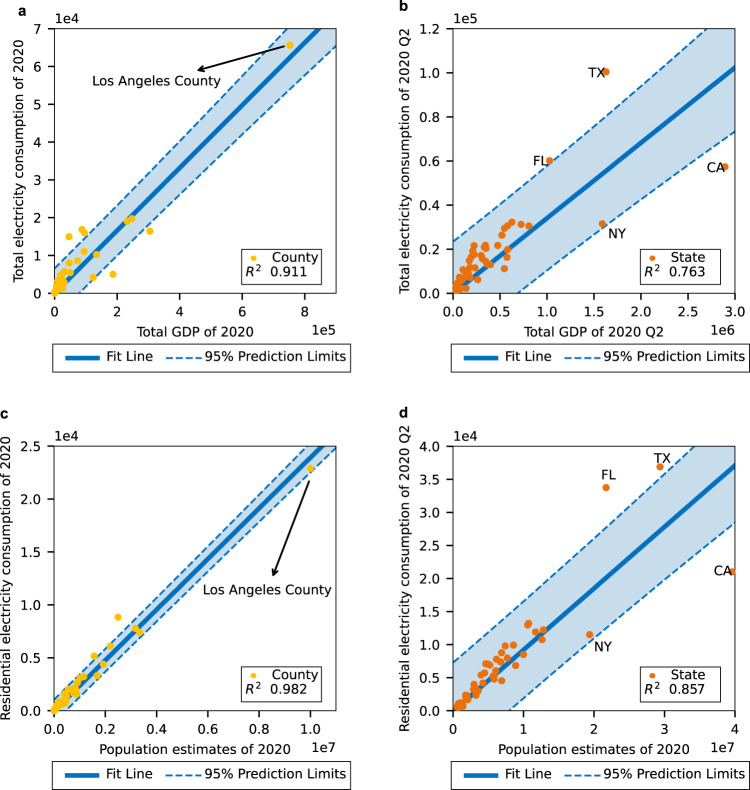
EC relationship with GDP and population. **a** Total EC and total GDP of each county in California in 2020. **b** Total EC and total GDP of each state in the U.S. in 2020 Q2. **c** Residential EC and population estimates of each county in California in 2020. **d** Residential EC in 2020 Q2 and population estimates for 2020 for each state in the U.S. The error bars of **a** to **d** represent 95% confidence prediction limits. The units of the *x* axes of **a** and **b** are millions of dollars, and the units of the *y* axes of **a** to **d** are GWh.

Figure 6 shows the EC against GDP and population. Figure 6a indicates that in 2019 in California, the county level total EC^44^ had a linear relationship with the total GDP^42^. Figure 6b indicates that in the second quarter of 2020, the total EC^45^ was linearly related with the total GDP^46^ at the state level in the continental US. Figure 6c shows that residential EC has a linear relationship with the population of each county in California in 2019. Figure 6d indicates that in the U.S., during the second quarter of 2020, state level residential EC could be roughly represented by the population of the year 2020^40^. From Fig. 6b, d, it can be observed that four states, namely Florida, Texas, New York, and California, deviate from the linear regression line. Further, the GDP and population range on the state level is much wider than the county level. As a result, inaccuracy can be introduced if we apply a linear regression model built from state level data to estimate county-level EC. To overcome the drawback mentioned above, a linear proportional model at the county level is applied for the following reasons. First, Fig. 6a (or 6c) indicates the linear relationship between total EC and GDP (or between residential EC and population) on the county level in California. Second, due to county-level data unavailability in other states, the linear proportional model with state-specific coefficients is applied at the county level in each state, which is modeled by Eqs. 3 and 4. Third, as such, the estimates on the county level within a state can avoid the impacts from other states.

GDP, EC, and population are closely related to each other, and both GDP and population can be an indicator of EC. As shown in Supplementary Table 5, the population outperforms the GDP in the ordinary least squares (OLS) regression analysis on the total/residential EC. However, given the widespread adoption of work-from-home policies, it is more prudent to use GDP and the intensity of information technology to measure total EC. Additionally, it can be observed that when both GDP and population are included in the model, the sign of the GDP coefficient becomes negative, which is consistent with the high degree of collinearity between GDP and population. The Pearson correlation coefficient between GDP and population at the county level in California and the state level are 0.9562 and 0.9753, respectively. Thus, the GDP is used to estimate the total EC, whereas the population is used to estimate the residential EC.

As a result, the county level total EC was estimated through the county GDP share in the state, while the residential EC was estimated based on the proportion of county population in the state. These are given in Eqs. (3) and (4):

3 $${{{{{{\rm{ECT}}}}}}}_{{{{{{\rm{c}}}}}}}=\frac{{{{{{{\rm{GDP}}}}}}}_{{{{{{\rm{c}}}}}}}}{{{{{{{\rm{GDP}}}}}}}_{{{{{{\rm{s}}}}}}}}{{{{{{\rm{ECT}}}}}}}_{{{{{{\rm{s}}}}}}}$$

in which ECT_c_ is the total EC of a county, GDP2_c_ is the annual GDP in current dollars of the county, GDP2_s_ is the annualized quarterly GDP in current dollars of the according state, and ECT_s_ is the total EC of the corresponding state.

4 $${{{{{{\rm{ECR}}}}}}}_{{{{{{\rm{c}}}}}}}=\frac{{P}_{{{{{{\rm{c}}}}}}}}{{P}_{{{{{{\rm{s}}}}}}}}{{{{{{\rm{ECR}}}}}}}_{{{{{{\rm{s}}}}}}}$$

in which ECR_c_ is the residential EC of a county, *P*_c_ is the population estimate of the county, *P*_s_ is the population estimate of the according state, and ECR_s_ is the residential EC of the corresponding state.

The estimates from April–May 2019 and April–May 2020 were constructed. Then, the estimates of metropolitan EC could be aggregated based on the county-level data:

5 $$\left\{\begin{array}{c}{{{{{{\rm{ECT}}}}}}}_{{{{{{\rm{MSA}}}}}},y}=\sum {{ECT}}_{{{{{{\rm{county}}}}}},y}\\ {{{{{{\rm{ECR}}}}}}}_{{{{{{\rm{MSA}}}}}},y}=\sum {{ECR}}_{{{{{{\rm{county}}}}}},y}\end{array}\right.$$

where *y* is the year, which can be either 2019 or 2020.

Therefore, the EC change after COVID-19 can be calculated as:

6 $$\left\{\begin{array}{c}{r}_{{{{{{\rm{ECT}}}}}}}=\frac{{{{{{{\rm{ECT}}}}}}}_{{{{{{\rm{MSA}}}}}},2020}-{{{{{{\rm{ECT}}}}}}}_{{{{{{\rm{MSA}}}}}},2019}}{{{{{{{\rm{ECT}}}}}}}_{{{{{{\rm{MSA}}}}}},2019}}\\ {r}_{{{{{{\rm{ECR}}}}}}}=\frac{{{{{{{\rm{ECR}}}}}}}_{{{{{{\rm{MSA}}}}}},2020}-{{{{{{\rm{ECR}}}}}}}_{{{{{{\rm{MSA}}}}}},2019}}{{{{{{{\rm{ECR}}}}}}}_{{{{{{\rm{MSA}}}}}},2019}}\end{array}\right.$$

In the second step, the GDP of 2020 was estimated. The 2019 GDP by county and 2019 Q2 to 2020 Q2 quarterly GDP by state were released by the U.S. Bureau of Economic Analysis^46^. However, the 2020 GDP by county was calculated from annualized quarterly GDP growth by state. It is assumed that GDP growth in April and May were consistent with the growth in Q2.

First, the base of the county GDP growth was measured by the chain-type quantity indexes for real GDP (inflation-adjusted) by state, as shown in (7),

7 $${\rho }_{{{\mbox{c,}}}2020}^{0}=\frac{{{\mbox{GDP}}}{8}_{{{\mbox{s,}}}2020{{\mbox{Q}}}2}}{{{\mbox{GDP}}}{8}_{{{\mbox{s,}}}2019{{\mbox{Q}}}2}}$$

in which the ${\rho }_{{{{{{\rm{c}}}}}},2020}^{0}$ is the base GDP growth rate of a county in 2020, and GDP8_s,2020Q2_ and GDP8_s,2019Q2_ are the annualized quarterly GDP chain-type quantity indexes for real GDP by the state in 2020 Q2 and 2019 Q2, respectively. Second, the GDP growth rate of each county was adjusted by the information technology intensity based on the assumption that the production of industries after COVID-19 was proportional to their intensity of information technology and that part of each county’s workforce could work from home^47^. The information technology intensity for each industry is shown in Supplementary Fig. 1. We imposed a discount factor on the GDP growth rate caused by the non-information industries as shown in (8):

8 $${\rho }_{{{\mbox{c,}}}2020}^{{{\mbox{adj}}}}=\frac{2}{12}\left[\left(1-{T}_{{{\mbox{MSA}}}}\right)-\left(1-{\bar{T}}_{{{\mbox{MSA}}}}\right)\right]$$

in which ${\rho }_{{{{{{\rm{c}}}}}},2020}^{{{{{{\rm{adj}}}}}}}$ is the penalty coefficient of the county, *T*_MSA_ is the information technology intensity gain of the corresponding MSA as shown in (9), and ${\bar{T}}_{{{{{{\rm{MSA}}}}}}}$ is the mean value of *T*_MSA_.

9 $${T}_{{{\mbox{MSA}}}}=\mathop{\sum }\limits_{{{{{{\rm{k}}}}}}=1}^{20}\frac{{{{{{{\rm{d}}}}}}}_{{{{{{\rm{k}}}}}}}{{\mbox{GDP}}}{2}_{{{{{{\rm{k}}}}}},{{\mbox{MSA}}}}}{{{\mbox{GDP}}}{2}_{{{\mbox{MSA}}}}}$$

where *d*_k_ is the percentage of digital workers by industry^47^, GDP2_*k*,MSA_ is the GDP in current dollars by industry of the MSA, and GDP2_MSA_ is the total GDP in current dollars of the MSA.

Finally, the 2020 GDP by county was calculated by the 2019 GDP and 2020 growth rate as:

10 $${{\mbox{GDP}}}{2}_{{{\mbox{c,}}}2020}=\left({\rho }_{{{\mbox{c,}}}2020}^{0}-{\rho }_{{{\mbox{c,}}}2020}^{{{\mbox{adj}}}}\right){{\mbox{GDP}}}{2}_{{{\mbox{c,}}}2019}$$

where GDP2_c,2020_ is the county GDP in 2020, and GDP2_c,2019_ is the county GDP in 2019.

### Data processing and computation

**Fig. 7:**
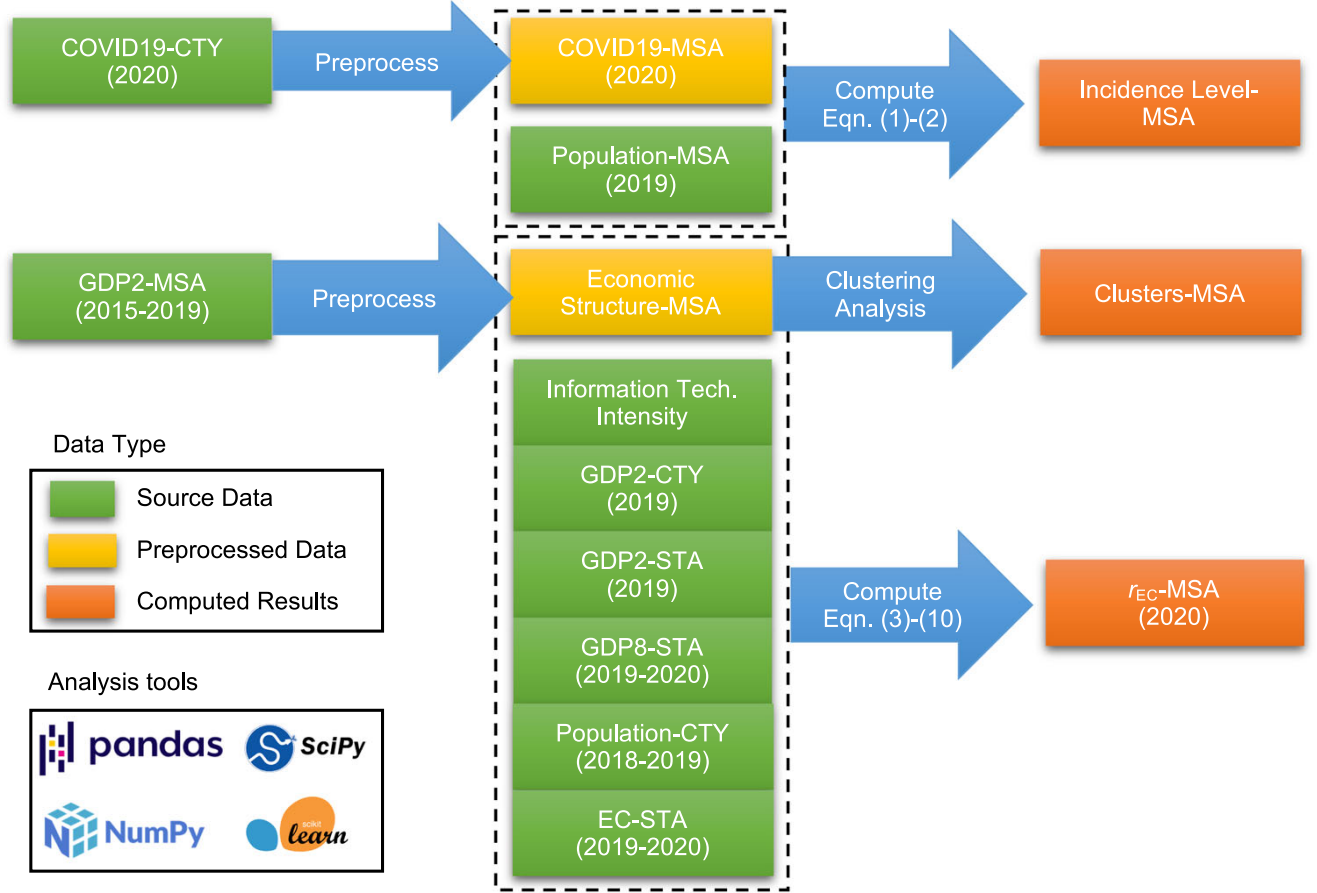
Data processing and computation flow. Green boxes denote the source data, yellow boxes denote preprocessed data, and orange boxes denote the computed results. The source data can be found in the reference, and a copy of the raw source data and computation results can be found in the Data availability section.

The data processing and computation flow is depicted in Fig. 7, where MSA stands for metropolitan statistical area, CTY for county, and STA for state. The green boxes denote the source data, which include COVID-19 data at the county level; GDP data at the county, metropolitan, and state levels; population data at the county, metropolitan, and state levels; EC data at the state level; and data on information technology intensity in the U.S. Yellow boxes denote preprocessed data, which includes COVID-19 and economic structure data at the metropolitan level, where missing value filling and data transformation are applied. Orange boxes denote the computed results, which include the level of incidence at the metropolitan level, metropolitan clusters, and EC and its variation at the metropolitan level. The source data were obtained in CSV format. Then the data were preprocessed (i.e., cleaned, aggregated, and transformed) and computed by NumPy^48^ and pandas^49^. The clustering analysis and other statistical analysis were performed with Scikit-learn^50^ and SciPy^51^. All the tools mentioned above are open-source and can be accessed by the public.

### Reporting summary

Further information on research design is available in the Nature Portfolio Reporting Summary linked to this article.

## Supplementary information

Supplementary Information

Reporting Summary

## Data Availability

The EC, economic structure, COVID data of MSAs generated in this study, and a copy of the raw data have been deposited in the figshare database at 10.6084/m9.figshare.20493345.v2. All the raw data can be accessed by the public at the referenced link.

## Code availability

All codes used in this research are available from the author upon request.

## References

[1] Bick, A. et al. Work from home before and after the Covid-19 outbreak. *SSRN*. (2021).

[2] U.S. Energy Information Administration (EIA). Low transportation fuel demand and low profitability drive refinery run declines. Today in Energy. https://www.eia.gov/todayinenergy/detail.php?id=43595# (2020).

[3] NYISO. NYISO Updates COVID-19 Demand Impacts. https://www.nyiso.com/-/press-release-7c-nyiso-details-covid-19-demand-impacts (2021).

[4] U.S. Energy Information Administration (EIA). EIA forecasts lower U.S. natural gas consumption in 2020. Today in Energy. https://www.eia.gov/todayinenergy/detail.php?id=43856 (2020).

[5] Liu Z (2020). Near-real-time monitoring of global CO2 emissions reveals the effects of the COVID-19 pandemic. Nat. Commun..

[6] Wang Q, Su M (2020). A preliminary assessment of the impact of COVID-19 on environment. A case study of China. Sci. Total Environ..

[7] Chakraborty I, Maity P (2020). COVID-19 outbreak: Migration, effects on society, global environment and prevention. Sci. Total Environ..

[8] Zambrano-Monserrate MA, Ruano MA, Sanchez-Alcalde L (2020). Indirect effects of COVID-19 on the environment. Sci. Total Environ..

[9] Bhat SA (2021). Impact of COVID-related lockdowns on environmental and climate change scenarios. Environ. Res..

[10] U.S. Energy Information Administration (EIA). Electric Power Monthly. https://www.eia.gov/electricity/monthly/index.php.

[11] U.S. Energy Information Administration (EIA). Annual Energy Outlook 2021. https://www.eia.gov/outlooks/aeo/tables_side.php (2021).

[12] U.S. Energy Information Administration (EIA). Renewables account for most new U.S. electricity generating capacity in 2021. Today in Energy. https://www.eia.gov/todayinenergy/detail.php?id=46416 (2021).

[13] American Council on Renewable Energy (ACORE). Expectations for Renewable Energy Finance in 2021-2024: growing Confidence in the Aftermath of the Pandemic. (2021).

[14] Tuohy, A., Kelly, A., Deaver, B., Lannoye, E. & Brooks, D. COVID-19 bulk system impacts demand impacts and operational and control center practices. 33 (EPRI Transmission Operations and Planning, 2020).

[15] Implications of COVID-19 for the electricity industry: a comprehensive review. *CSEE JPES.*10.17775/CSEEJPES.2020.02500 (2020).

[16] Paaso, A. et al. Sharing knowledge on electrical energy industry’s first response to COVID19. (2020).

[17] U.S. Energy Information Administration (EIA). Daily electricity demand impacts from COVID-19 mitigation efforts differ by region. Today in Energy. https://www.eia.gov/todayinenergy/detail.php?id=43636 (2020).

[18] Ruan, G. et al. A cross-domain approach to analyzing the short-run impact of COVID-19 on the U.S. electricity sector. https://arxiv.org/abs/2005.06631 (2020). 10.1016/j.joule.2020.08.017 PMC7523140 33015556

[19] Ferguson R, Wilkinson W, Hill R (2000). Electricity use and economic development. Energy Policy.

[20] U.S. Energy Information Administration (EIA). U.S. economy and electricity demand growth are linked, but relationship is changing. Today in Energy. https://www.eia.gov/todayinenergy/detail.php?id=10491 (2013).

[21] Arora V, Lieskovsky J. Electricity use as an indicator of US economic activity. (2016).

[22] Apergis N, Payne JE (2011). A dynamic panel study of economic development and the electricity consumption-growth nexus. Energy Econ..

[23] Zhang C, Zhou K, Yang S, Shao Z (2017). On electricity consumption and economic growth in China. Renew. Sustain. Energy Rev..

[24] Al-Bajjali SK, Shamayleh AY (2018). Estimating the determinants of electricity consumption in Jordan. Energy.

[25] Yu H (2012). The influential factors of China’s regional energy intensity and its spatial linkages: 1988–2007. Energy Policy.

[26] An H, Xu J, Ma X (2020). Does technological progress and industrial structure reduce electricity consumption? Evidence from spatial and heterogeneity analysis. Struct. Chang. Econ. Dyn..

[27] US Census Bureau. U.S. Census Bureau’s geographic spatial data. https://www.census.gov/geographies/mapping-files.html.

[28] California Energy Commission. Energy Commission Releases New Data on How COVID-19 is Impacting the Energy Sector. https://www.energy.ca.gov/news/2020-05/energy-commission-releases-new-data-how-covid-19-impacting-energy-sector (2020).

[29] MISO. COVID-19 Impacts to MISO Load and Outage Coordination. https://cdn.misoenergy.org/COVID%2019%20Impacts%20to%20MISO%20Load%20and%20Outage_as%20of%20June20454548.pdf (2020).

[30] PJM. PJM Updates Pandemic Impacts to Current Load, Long-Term Forecast. PJM Inside Lines. https://insidelines.pjm.com/pjm-updates-pandemic-impacts-to-current-load-long-term-forecast/ (2020).

[31] Cabral JDA, Legey LFL, Freitas Cabral MVD (2017). Electricity consumption forecasting in Brazil: a spatial econometrics approach. Energy.

[32] Yukseltan E, Yucekaya A, Bilge AH (2017). Forecasting electricity demand for Turkey: modeling periodic variations and demand segregation. Appl. Energy.

[33] Jasiński T (2019). Modeling electricity consumption using nighttime light images and artificial neural networks. Energy.

[34] Cao G, Wu L (2016). Support vector regression with fruit fly optimization algorithm for seasonal electricity consumption forecasting. Energy.

[35] Wang S, Sun X, Lall U (2017). A hierarchical Bayesian regression model for predicting summer residential electricity demand across the U.S.A. Energy.

[36] Bianco V, Manca O, Nardini S (2009). Electricity consumption forecasting in Italy using linear regression models. Energy.

[37] Sarwar S, Chen W, Waheed R (2017). Electricity consumption, oil price and economic growth: global perspective. Renew. Sustain. Energy Rev..

[38] Filipović S, Verbič M, Radovanović M (2015). Determinants of energy intensity in the European Union: a panel data analysis. Energy.

[39] Covid Act Now. U.S. COVID Risk & Vaccine Tracker. https://covidactnow.org.

[40] US Census Bureau. County Population Totals: 2010-2019. https://www.census.gov/data/datasets/time-series/demo/popest/2010s-counties-total.html.

[41] COVID-19 US County JHU Data & Demographics. https://kaggle.com/headsortails/covid19-us-county-jhu-data-demographics.

[42] U.S. Bureau of Economic Analysis (BEA). GDP by County, Metro, and Other Areas. https://www.bea.gov/data/gdp/gdp-county-metro-and-other-areas.

[43] Han, J. & Kamber, M. *Data mining: concepts and techniques*. (Elsevier; Morgan Kaufmann, 2006).

[44] California Energy Commission. Electricity Consumption by County. https://ecdms.energy.ca.gov/elecbycounty.aspx.

[45] Energy Information Administration (EIA). Electricity data browser. https://www.eia.gov/electricity/data/browser/.

[46] U.S. Bureau of Economic Analysis (BEA). GDP by State. https://www.bea.gov/data/gdp/gdp-state.

[47] Makridis, C. & Hartley, J. The cost of Covid-19: a rough estimate of the 2020 US GDP impact. *SSRN J.*10.2139/ssrn.3570731 (2020).

[48] Harris CR (2020). Array programming with NumPy. Nature.

[49] McKinney, W. Data structures for statistical computing in python. In *Proceedings of the 9th Python in Science Conference* **445**, 51–56 10.25080/Majora-92bf1922-00a (2010).

[50] Pedregosa, Fabian, et al. Scikit-learn: Machine learning in Python. *J. Mach. Learn. Res.* **12**, 2825–2830 (2011).

[51] Virtanen P (2020). SciPy 1.0: fundamental algorithms for scientific computing in Python. Nat. Methods.

