# Supplementary material for: Electricity consumption variation versus economic structure during COVID-19 on metropolitan statistical areas in the US
Source: Nat Commun. 2022 Nov 19;13:7122. doi: 10.1038/s41467-022-34447-7 (PMC9675752; doi:10.1038/s41467-022-34447-7)
Supplement: Supplementary file 1 — Supplementary Information [file 41467_2022_34447_MOESM1_ESM.docx]

# Supplementary Information to *Electricity Consumption Variation versus Economic Structure during COVID-19 on Metropolitan Statistical Areas in the US*

Jinning Wang, Fangxing Li, Hantao Cui, Qingxin Shi, Trey Mingee

## Supplementary Notes

### Supplementary Note 1 k-means for economic structure clustering analysis

The main idea of k-means ^R1^ is to classify the observations into several clusters by minimizing the total inertia *J* as defined in equation (1), which is the sum of squares of the distance between observations and the centroids of all clusters. In equation (1), $x_{i}$ is an *n*-dimension (*n*=20 in this study) vector that represents the economic structure of the MSA, and it belongs to the $c_{i}$th cluster. Also, $\mu_{c_{i}}$ is the *n*-dimension vector as the centroid of the $c_{i}$th cluster. The initialized cluster centers are randomly selected, then the centers are updated as the arithmetic mean of the points within the cluster. The procedures of k-means can be summarized as:

1. For a given number of clusters:
2. Initialize the cluster centroids with randomly selected points.
3. Classify each observation to the closest cluster centroid.
4. Compute the new cluster centroids by the arithmetic mean of all observations within the cluster and compute the total inertia with equation (1).
5. Repeat b)-c) until either the maximum number of iterations or the error tolerance is reached.

2) Test different numbers of clusters by repeating step 1), determine the number of clusters by the elbow method, such that the total inertia does not decrease much when the number of clusters is increased.

The outcome of k-means clustering analysis is the cluster centroids and cluster label for each observation. In this study, the cluster centroids represent the typical economic structure of the MSAs within the cluster.

$J(\mu)=\sum_{i=1}^{N} ||x_{i}-\mu_{c_{i}}||^{2}$ (1)

## Supplementary Figures


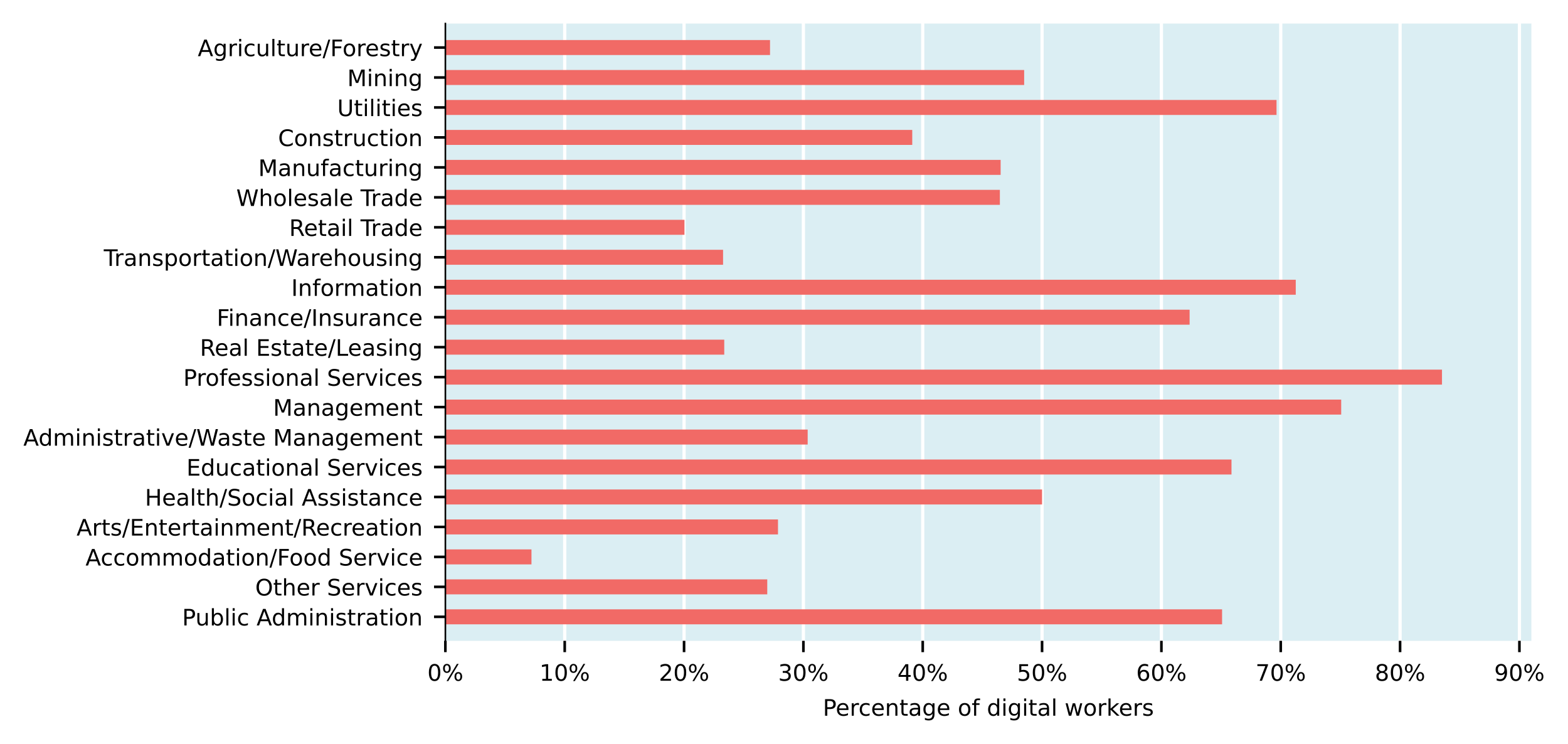


### Supplementary Fig. 1 Intensity of information technology for each industry

## Supplementary Tables

Supplementary Table 1 Results of two-sided Wilcoxon rank sum test of economic structure of updated version versus previous version of MSA-GDP GDP data. The sample sizes (*n_1_*, and *n_2_*) of each test are 44-44, 19-18, 110-123, 42-46, 120-104, 13-12, 16-16, 5-7, respectively.

| Cluster | Agriculture/ Forestry | Mining | Utilities | Construction | Manufacturing |
| --- | --- | --- | --- | --- | --- |
| I | 0.7385 | 0.5991 | 0.8938 | 0.6583 | 0.7260 |
| II | 0.5234 | 0.5844 | 0.9758 | 0.8316 | 0.3949 |
| III | 0.2985 | 1.0000 | 0.2730 | 0.3852 | 0.2043 |
| IV | 0.1813 | 0.9534 | 0.3538 | 0.8673 | 0.9933 |
| V | 0.3385 | 0.5123 | 0.4530 | 0.3729 | 0.9177 |
| VI | 0.1573 | 0.2314 | 0.1739 | 0.6635 | 0.7442 |
| VII | 0.4739 | 0.5718 | 0.7630 | 0.7063 | 0.7345 |
| VIII | 0.1675 | 0.9353 | 0.1229 | 0.8075 | 0.8075 |
| Cluster | Wholesale trade | Retail trade | Transportation/  Warehousing | Information | Finance/  Insurance |
| I | 0.5205 | 0.7011 | 0.3457 | 0.2854 | 0.8740 |
| II | 0.2482 | 0.5844 | 0.4120 | 0.1715 | 0.5038 |
| III | 0.5180 | 0.9286 | 0.9953 | 0.6713 | 0.9798 |
| IV | 0.2102 | 0.8021 | 0.7572 | 0.8476 | 0.6946 |
| V | 0.6463 | 0.1114 | 0.7518 | 0.4860 | 0.4249 |
| VI | 0.5139 | 0.9134 | 0.7856 | 0.4146 | 0.3841 |
| VII | 0.7919 | 0.5465 | 0.8505 | 0.8802 | 1.0000 |
| VIII | 0.0882 | 0.5698 | 0.2232 | 0.5698 | 0.4649 |
| Cluster | Real estate/ Leasing | Professional services | Management | Administrative services | Educational services |
| I | 0.3414 | 0.6949 | 0.9070 | 0.7512 | 0.8023 |
| II | 0.9758 | 0.5038 | 0.7845 | 0.7612 | 0.5844 |
| III | 0.1888 | 0.4113 | 0.9194 | 0.4773 | 0.7013 |
| IV | 0.2355 | 0.8607 | 0.7320 | 0.9467 | 0.8871 |
| V | 0.5867 | 0.6884 | 0.6687 | 0.9177 | 0.5406 |
| VI | 0.8704 | 0.7034 | 0.6245 | 0.4795 | 0.8704 |
| VII | 0.8505 | 0.8802 | 0.7630 | 0.6785 | 0.7630 |
| VIII | 0.4649 | 0.8075 | 0.6847 | 0.4649 | 0.6847 |
| Cluster | Health/Social assistance | Arts/Entertainment/  Recreation | Accommodation/  Food services | Other services | Public administration |
| I | 0.9202 | 0.7959 | 0.8478 | 0.6887 | 0.7766 |
| II | 0.6485 | 0.1212 | 0.5234 | 1.0000 | 0.7612 |
| III | 0.8978 | 0.8870 | 0.3461 | 0.2106 | 0.9907 |
| IV | 0.8542 | 0.3669 | 0.4572 | 0.5201 | 0.8215 |
| V | 0.6256 | 0.4333 | 0.7659 | 0.4060 | 0.7175 |
| VI | 0.7034 | 0.2767 | 0.7034 | 0.6635 | 0.6245 |
| VII | 0.8802 | 0.5718 | 0.5977 | 0.4739 | 0.7630 |
| VIII | 0.5698 | 0.2232 | 0.3718 | 0.3718 | 0.3718 |

Supplementary Table 2 Results of two-sided Wilcoxon rank sum test of EC on MSA level of updated version versus previous version. The sample sizes (*n_1_*, and *n_2_*) of each test are 44-44, 19-18, 110-123, 42-46, 120-104, 13-12, 16-16, 5-7, respectively.

| Cluster | | I | II | III | IV | V | VI | VII | VIII |
| --- | --- | --- | --- | --- | --- | --- | --- | --- | --- |
| Total EC | *W* | -2.5203 | -0.8812 | -1.8768 | -1.9632 | -2.3959 | -0.1632 | -0.7161 | -1.7052 |
|  | *p*-value | *0.0117 | 0.3782 | 0.0605 | *0.0496 | *0.0166 | 0.8704 | 0.4739 | 0.0882 |
| Residential EC | W | -0.4173 | -0.5774 | -0.4400 | -0.8354 | -0.6698 | 1.3598 | -0.7538 | -1.5428 |
|  | *p*-value | 0.6765 | 0.5637 | 0.6599 | 0.4035 | 0.5030 | 0.1739 | 0.4510 | 0.1229 |

### Supplementary Table 3 Results of two-sided Wilcoxon rank sum test of EC between different COVID-19 incidence levels.

| Category | Total EC variation | | Residential EC variation | |
| --- | --- | --- | --- | --- |
| Incidence | *W* | *p*-value | *W* | *p*-value |
| Low-Medium (*n_1_*=63, *n_2_*=62) | 3.3868 | **0.0007 | 0.2324 | 0.8162 |
| Low-High  (*n_1_*=272, *n_2_*=273) | 1.9658 | *0.0493 | -1.5348 | 0.1248 |
| Medium-High (*n_1_*=11, *n_2_*=10) | -0.4934 | 0.6217 | -1.6714 | 0.0946 |

### Supplementary Table 4 Snapshot of the source data and preprocessed economic structure data.

| Asheville, NC | Source Data (thousands of current dollars) | | | | | Preprocessed Data | |
| --- | --- | --- | --- | --- | --- | --- | --- |
| GDP Categories | 2015 | 2016 | 2017 | 2018 | 2019 | Scaled | Transformed |
| Agriculture/Forestry | 116,903 | 110,383 | 116,185 | (D) | (D) | 0.0053 | 0.3506 |
| Mining | 21,944 | 20,216 | 19,525 | 21,007 | 21,271 | 0.001 | 0.2505 |
| Utilities | (D) | (D) | (D) | 194,981 | (D) | 0.009 | 0.3902 |
| Construction | 691,476 | 767,455 | 840,363 | 952,477 | 1,026,595 | 0.0476 | 0.544 |
| Manufacturing | 2,932,732 | 2,890,797 | 3,199,748 | 3,426,440 | 3,690,299 | 0.1694 | 0.7011 |
| Wholesale trade | (D) | (D) | (D) | (D) | (D) | 0.0465 | 0.5413 |
| Retail trade | 1,272,478 | 1,344,928 | 1,409,664 | 1,476,397 | 1,551,513 | 0.072 | 0.5906 |
| Transportation/Warehousing | 379,347 | 337,175 | 357,999 | (D) | 433,586 | 0.0201 | 0.4579 |
| Information | 374,329 | 391,701 | 375,426 | 376,200 | 398,292 | 0.0185 | 0.4502 |
| Finance/Insurance | 808,779 | 850,903 | 919,992 | 1,037,836 | 1,030,865 | 0.0478 | 0.5444 |
| Real estate/Leasing | 2,400,763 | 2,512,960 | 2,704,360 | 2,815,130 | 2,993,489 | 0.1389 | 0.6738 |
| Professional services | (D) | (D) | (D) | (D) | (D) | 0.0465 | 0.5413 |
| Management | (D) | (D) | (D) | (D) | (D) | 0.0465 | 0.5413 |
| Administrative services | 546,412 | 565,953 | 577,267 | 616,706 | 662,860 | 0.0308 | 0.4984 |
| Educational services | (D) | (D) | (D) | (D) | (D) | 0.0465 | 0.5413 |
| Health/Social assistance | (D) | (D) | (D) | (D) | (D) | 0.0465 | 0.5413 |
| Art/Entertainment/Recreation | 190,443 | 210,737 | 225,000 | 269,295 | 285,066 | 0.0132 | 0.421 |
| Accommodation/Food services | 893,646 | 980,131 | 1,057,153 | 1,087,696 | 1,160,874 | 0.0539 | 0.9575 |
| Other services | 482,466 | 510,583 | 531,901 | 581,032 | 641,029 | 0.0297 | 0.4951 |
| Public administration | 1,988,924 | 2,033,363 | 2,137,348 | 2,267,588 | 2,377,729 | 0.1103 | 0.6435 |

### Supplementary Table 5 Ordinary least squared regression results. The data are scaled using the Min-Max method and the regression’s intercept is set to 0. The statistical tests for the coefficient are two-sided.

| Category | | | Total EC | | | | | Residential EC | | | | |
| --- | --- | --- | --- | --- | --- | --- | --- | --- | --- | --- | --- | --- |
| Level | Model | Vars. | coef. | σ | *p* | *R^2^* | df | coef. | σ | *p* | *R^2^* | df |
| County | 1 | GDP | 0.9493 | 0.0390 | ****<1e-4 | 0.9110 | 57 | 0.9608 | 0.0490 | ****<1e-4 | 0.8730 | 57 |
|  | 2 | Population | 1.0105 | 0.0180 | ****<1e-4 | 0.9820 | 57 | 1.0446 | 0.0190 | ****<1e-4 | 0.9820 | 57 |
|  | 3 | GDP | -0.0023 | 0.0670 | 0.9730 | 0.9820 | 56 | -0.2875 | 0.0580 | ****<1e-4 | 0.9870 | 56 |
|  |  | Population | 1.0127 | 0.0690 | ****<1e-4 |  |  | 1.3286 | 0.0590 | ****<1e-4 |  |  |
| State | 1 | GDP | 0.9701 | 0.0760 | ****<1e-4 | 0.7630 | 50 | 1.0383 | 0.0920 | ****<1e-4 | 0.7170 | 50 |
|  | 2 | Population | 0.9201 | 0.0470 | ****<1e-4 | 0.8860 | 50 | 0.9992 | 0.0580 | ****<1e-4 | 0.8570 | 50 |
|  | 3 | GDP | -1.6380 | 0.1670 | ****<1e-4 | 0.9610 | 49 | -2.2172 | 0.1630 | ****<1e-4 | 0.9700 | 49 |
|  |  | Population | 2.3369 | 0.1470 | ****<1e-4 |  |  | 2.9171 | 0.1440 | ****<1e-4 |  |  |
