# Supplementary material for: Electricity consumption variation versus economic structure during COVID-19 on metropolitan statistical areas in the US
Source: Nat Commun. 2022 Nov 19;13:7122. doi: 10.1038/s41467-022-34447-7 (PMC9675752; doi:10.1038/s41467-022-34447-7)
Supplement: Supplementary file 2 — Reporting Summary [file 41467_2022_34447_MOESM2_ESM.pdf]

nature portfolio

Corresponding author(s): Fangxing Li

Last updated by author(s): Oct 18, 2022

Reporting Summary

Nature Portfolio wishes to improve the reproducibility of the work that we publish. This form provides structure for consistency and transparency in reporting. For further information on Nature Portfolio policies, see our Editorial Policies and the Editorial Policy Checklist.

Statistics

For all statistical analyses, confirm that the following items are present in the figure legend, table legend, main text, or Methods section.

n/a	Confirmed	
☐	☒	The exact sample size (n) for each experimental group/condition, given as a discrete number and unit of measurement
☒	☐	A statement on whether measurements were taken from distinct samples or whether the same sample was measured repeatedly
☐	☒	The statistical test(s) used AND whether they are one- or two-sided<br>*Only common tests should be described solely by name; describe more complex techniques in the Methods section.*
☐	☒	A description of all covariates tested
☐	☒	A description of any assumptions or corrections, such as tests of normality and adjustment for multiple comparisons
☐	☒	A full description of the statistical parameters including central tendency (e.g. means) or other basic estimates (e.g. regression coefficient) AND variation (e.g. standard deviation) or associated estimates of uncertainty (e.g. confidence intervals)
☐	☒	For null hypothesis testing, the test statistic (e.g. F, t, r) with confidence intervals, effect sizes, degrees of freedom and P value noted<br>*Give P values as exact values whenever suitable.*
☒	☐	For Bayesian analysis, information on the choice of priors and Markov chain Monte Carlo settings
☒	☐	For hierarchical and complex designs, identification of the appropriate level for tests and full reporting of outcomes
☐	☒	Estimates of effect sizes (e.g. Cohen's d, Pearson's r), indicating how they were calculated

Our web collection on statistics for biologists contains articles on many of the points above.

Software and code

Policy information about availability of computer code

Data collection	All data sets are collected from publicly available sources including US Energy Information Administration (EIA), American Council on Renewable Energy (ACORE), US Census Bureau, U.S. Bureau of Economic Analysis, U.S. COVID Risk & Vaccine Tracker - COVID Act Now (https://covidactnow.org), COVID-19 US County JHU Data & Demographics, California Energy Commission, NYISO (New York Independent System Operator), MISO (Midcontinent Independent System Operator), and PJM. All relevant data sources are clearly and immediately cited in the paper when mentioned, and listed in the references.
Data analysis	All data pre-processing (i.e., cleaning, aggregation, and transformation) were performed by NumPy and pandas. The clustering analysis and other statistical analysis were performed with Scikit-learn and SciPy. All the tools mentioned above are open-source and can be accessed by the public. The above statement can also be found at the end of the first paragraph on page 14 on the submitted manuscript.

For manuscripts utilizing custom algorithms or software that are central to the research but not yet described in published literature, software must be made available to editors and reviewers. We strongly encourage code deposition in a community repository (e.g. GitHub). See the Nature Portfolio guidelines for submitting code & software for further information.

Data

Policy information about availability of data

All manuscripts must include a data availability statement. This statement should provide the following information, where applicable:

- Accession codes, unique identifiers, or web links for publicly available datasets
- A description of any restrictions on data availability
- For clinical datasets or third party data, please ensure that the statement adheres to our policy

All data analyzed in this study can be accessed from public resources (this statement is included near the end of the manuscript). All relevant data sources are clearly and immediately cited in the paper when mentioned, and listed in the references (also see our response to "Data Collection").

Field-specific reporting

Please select the one below that is the best fit for your research. If you are not sure, read the appropriate sections before making your selection.

☐ Life sciences ☐ Behavioural & social sciences ☒ Ecological, evolutionary & environmental sciences

For a reference copy of the document with all sections, see nature.com/documents/nr-reporting-summary-flat.pdf

Ecological, evolutionary & environmental sciences study design

All studies must disclose on these points even when the disclosure is negative.

Study description	This study is to discover the correlation between electricity consumption and economical structure under the initial months of COVID-19 breakout in metropolitan areas.
Research sample	Research samples include economic data and electricity consumption in the US, as well as the first two months COVID-19 cases. We choose to study the first two months of COVID breakout because the initial impact is always the most significant and the society and the electric industry was not prepared for such socio-economic crisis. Also, we choose the Metropolitan Statistical Areas (MSAs) in the U.S. because they account for 86% of the total population and 87% of the total electricity consumption.
Sampling strategy	Not applicable.
Data collection	Data sets were collected from publicly available sources as detailed in the paper whenever mentioned and cited immediately.
Timing and spatial scale	The two months window of April and May in 2019 and 2020 for comparison study.
Data exclusions	The estimates cover 380 Metropolitan Statistical Areas (MSAs) in the continental U.S. out of the total 384 MSAs rigorously defined by the United States Office of Management and Budget. In other words, this study does not cover the remaining 4 MSAs located in Hawaii and Alaska because they are not part of the continental US. The development of COVID-19, the economic structure and the electricity consumption may not be similar to that of the continental US.
Reproducibility	All data sets are real and actual data publicly available so other researchers can easily reproduce the results in our study. Also, similar approaches can be applied to potential or future socio-economic crisis.
Randomization	Not applicable.
Blinding	Not applicable.

Did the study involve field work? ☐ Yes ☒ No

Reporting for specific materials, systems and methods

We require information from authors about some types of materials, experimental systems and methods used in many studies. Here, indicate whether each material, system or method listed is relevant to your study. If you are not sure if a list item applies to your research, read the appropriate section before selecting a response.

Materials & experimental systems

n/a	Involved in the study
☒	☐ Antibodies
☒	☐ Eukaryotic cell lines
☒	☐ Palaeontology and archaeology
☒	☐ Animals and other organisms
☒	☐ Human research participants
☒	☐ Clinical data
☒	☐ Dual use research of concern

Methods

n/a	Involved in the study
☒	☐ ChIP-seq
☒	☐ Flow cytometry
☒	☐ MRI-based neuroimaging
